# *N*-(2-Phen­oxy­phen­yl)pyrazine-2-carboxamide

**DOI:** 10.1107/S1600536812041050

**Published:** 2012-10-20

**Authors:** Mehri Noroozi Tisseh, Maryam Kargar Razi, Hamid Reza Khavasi

**Affiliations:** a Department of Chemistry, Islamic Azad University, North Tehran Branch, Tehran, Iran; b Department of Chemistry, Shahid Beheshti University, G. C., Evin, Tehran, 1983963113, Iran

## Abstract

In the title compound, C_17_H_13_N_3_O_2_, the pyrazine ring is oriented at 1.65 (11) and 88.33 (17)° with respect to the benzene rings. The benzene rings are nearly perpendicular to each other [dihedral angle 87.14 (17)°]. In the crystal, a weak C—H⋯N hydrogen bond occurs.

## Related literature

For related structures, see: Wardell *et al.* (2008 ▶); de Lima Ferreira *et al.* (2010 ▶).
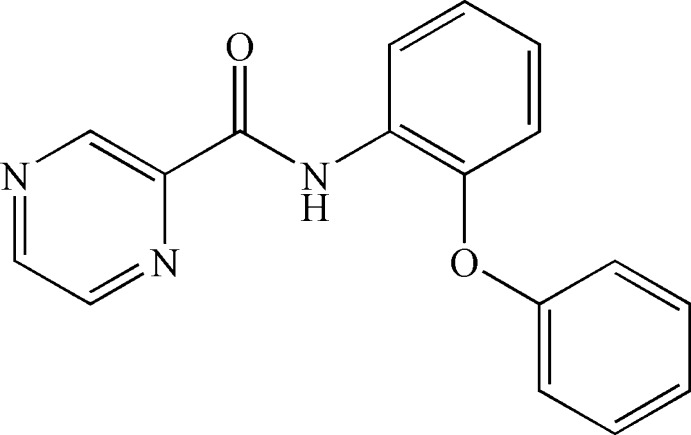


## Experimental

### 

#### Crystal data

C_17_H_13_N_3_O_2_
*M* *_r_* = 291.30
Triclinic, 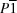

*a* = 5.0913 (10) Å
*b* = 11.769 (2) Å
*c* = 12.268 (3) Å
α = 91.058 (16)°
β = 94.541 (16)°
γ = 101.648 (15)°
*V* = 717.3 (3) Å^3^
*Z* = 2
Mo *K*α radiation
μ = 0.09 mm^−1^
*T* = 298 K
0.40 × 0.20 × 0.15 mm

#### Data collection

Stoe IPDS II diffractometer
5966 measured reflections
2811 independent reflections
1923 reflections with *I* > 2σ(*I*)
*R*_int_ = 0.055

#### Refinement

*R*[*F* ^2^ > 2σ(*F* ^2^)] = 0.062
*wR*(*F* ^2^) = 0.132
*S* = 1.07
2811 reflections
199 parameters
H-atom parameters constrained
Δρ_max_ = 0.13 e Å^−3^
Δρ_min_ = −0.16 e Å^−3^

### 

Data collection: *X-AREA* (Stoe & Cie, 2005 ▶); cell refinement: *X-AREA*; data reduction: *X-RED* (Stoe & Cie, 2005 ▶); program(s) used to solve structure: *SHELXS97* (Sheldrick, 2008 ▶); program(s) used to refine structure: *SHELXL97* (Sheldrick, 2008 ▶); molecular graphics: *ORTEP-3 for Windows* (Farrugia, 1997 ▶); software used to prepare material for publication: *WinGX* (Farrugia, 1999 ▶).

## Supplementary Material

Crystal structure: contains datablock(s) I, global. DOI: 10.1107/S1600536812041050/xu5626sup1.cif

Structure factors: contains datablock(s) I. DOI: 10.1107/S1600536812041050/xu5626Isup2.hkl

Supplementary material file. DOI: 10.1107/S1600536812041050/xu5626Isup3.cml

Additional supplementary materials:  crystallographic information; 3D view; checkCIF report

## Figures and Tables

**Table 1:** Hydrogen-bond geometry (Å, °)

*D*—H⋯*A*	*D*—H	H⋯*A*	*D*⋯*A*	*D*—H⋯*A*
C2—H2⋯N2^i^	0.93	2.62	3.439 (3)	147

Symmetry code: (i) 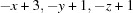.

**Fig. 1.**
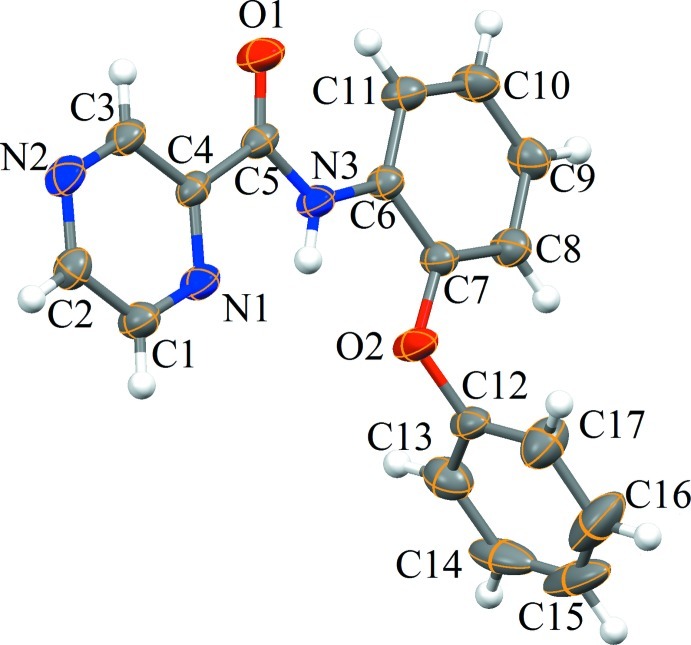
The molecular structure of the title molecule, with the atom-numbering scheme. Displacement ellipsoids are drawn at the 30% probability level. Hydrogen bonds are shown as dashed lines.

**Fig. 2.**
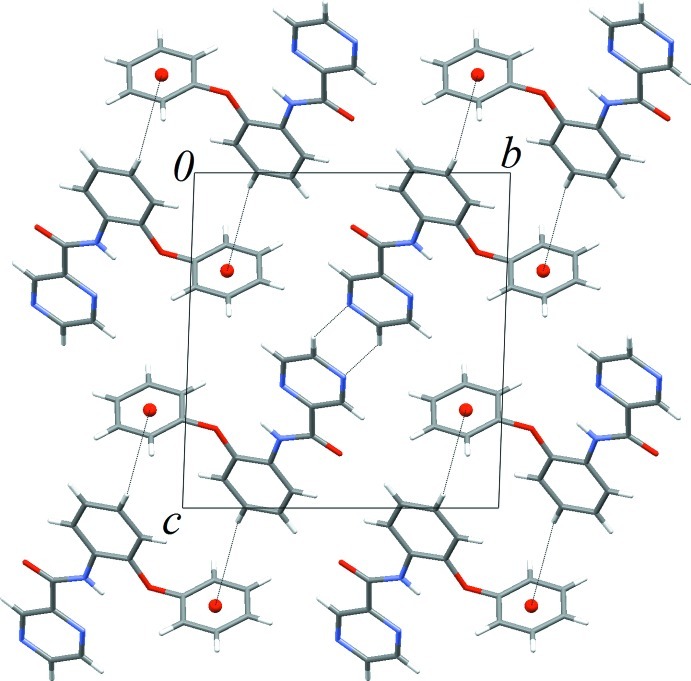
Unit-cell packing diagram for title molecule in a-direction. Hydrogen bonds and C—H···pi interactions are shown as dashed lines.

## supplementary crystallographic information

### Comment

The carboxamide [C(O)NH] group, ubiquitous throughout the nature in the primary structure of proteins, is an important ligand construction unit for coordination chemists. Pyrazine carboxamides are available from condensation reactions between pyrazine acid and amines, promoted by coupling agents such as triphenylphosphite (Wardell *et al.* (2008); de Lima Ferreira *et al.* (2010)). As part of our ongoing studies in this area, we report herein the crystal structure of the title compound, (I).

In the molecule of (I) (Fig. 1), the pyrazine ring is oriented with respect to the two benzene rings at 1.65 (11) and 88.33 (17)°, respectively, and the two benzenerings are nearly perpendicular to each other [dihedral angle 87.14 (17)°]. In the crystal structure, intermolecular C—H···N non-classical hydrogen bonds (Table 1, Fig. 2) may stabilize the structure.

### Experimental

2-Pyridinedicarboxylic acid (0.124 g, 1 mmol) was suspended in pyridine (10 ml). 8-aminoquinoline (0.182 g, 1 mmol) was added to the mixture, and the mixture was stirred at 313–318 K, for 10 min. Triphenylphosphite (2 mmol, 0..53 ml) was added dropwise to the resulting solution. The temperature of the reaction mixture was increased to 363–373 K, and the mixture was magnetically stirred for 4 h. After cooling to room temperature, the reaction mixture was left in the hood for 24 h. The white precipitate was filtered off. Recrystallization was achieved by diethyl ether diffusion into a chloroform solution of the compound at room temperature (yield; 78%, m.p. 550 K).

### Refinement

H atoms were positioned geometrically with N—H = 0.86 and C—H = 0.93 Å, and constrained to ride on their parent atoms with *U*_iso_(H) = 1.2*U*_eq_(N,C).

### Crystal data

**Table:**

C_17_H_13_N_3_O_2_	*Z* = 2
*M**_r_* = 291.30	*F*(000) = 304
Triclinic, *P*1	*D*_x_ = 1.349 Mg m^−^^3^
Hall symbol: -P 1	Mo *K*α radiation, λ = 0.71073 Å
*a* = 5.0913 (10) Å	Cell parameters from 5966 reflections
*b* = 11.769 (2) Å	θ = 2.4–26.0°
*c* = 12.268 (3) Å	µ = 0.09 mm^−^^1^
α = 91.058 (16)°	*T* = 298 K
β = 94.541 (16)°	Block, yellow
γ = 101.648 (15)°	0.40 × 0.20 × 0.15 mm
*V* = 717.3 (3) Å^3^	

### Data collection

**Table:**

Stoe IPDS II diffractometer	1923 reflections with *I* > 2σ(*I*)
Radiation source: fine-focus sealed tube	*R*_int_ = 0.055
Graphite monochromator	θ_max_ = 26.0°, θ_min_ = 2.4°
rotation method scans	*h* = −6→6
5966 measured reflections	*k* = −14→13
2811 independent reflections	*l* = −15→15

### Refinement

**Table:**

Refinement on *F*^2^	Primary atom site location: structure-invariant direct methods
Least-squares matrix: full	Secondary atom site location: difference Fourier map
*R*[*F*^2^ > 2σ(*F*^2^)] = 0.062	Hydrogen site location: inferred from neighbouring sites
*wR*(*F*^2^) = 0.132	H-atom parameters constrained
*S* = 1.07	*w* = 1/[σ^2^(*F*_o_^2^) + (0.0432*P*)^2^ + 0.2101*P*] where *P* = (*F*_o_^2^ + 2*F*_c_^2^)/3
2811 reflections	(Δ/σ)_max_ = 0.002
199 parameters	Δρ_max_ = 0.13 e Å^−^^3^
0 restraints	Δρ_min_ = −0.16 e Å^−^^3^

### Special details

**Table:**

Geometry. All e.s.d.'s (except the e.s.d. in the dihedral angle between two l.s. planes) are estimated using the full covariance matrix. The cell e.s.d.'s are taken into account individually in the estimation of e.s.d.'s in distances, angles and torsion angles; correlations between e.s.d.'s in cell parameters are only used when they are defined by crystal symmetry. An approximate (isotropic) treatment of cell e.s.d.'s is used for estimating e.s.d.'s involving l.s. planes.
Refinement. Refinement of *F*^2^ against ALL reflections. The weighted *R*-factor *wR* and goodness of fit *S* are based on *F*^2^, conventional *R*-factors *R* are based on *F*, with *F* set to zero for negative *F*^2^. The threshold expression of *F*^2^ > σ(*F*^2^) is used only for calculating *R*-factors(gt) *etc*. and is not relevant to the choice of reflections for refinement. *R*-factors based on *F*^2^ are statistically about twice as large as those based on *F*, and *R*- factors based on ALL data will be even larger.

### Fractional atomic coordinates and isotropic or equivalent isotropic displacement parameters (Å^2^)

**Table:**

	*x*	*y*	*z*	*U*_iso_*/*U*_eq_	
C1	1.1804 (5)	0.6963 (2)	0.4406 (2)	0.0618 (7)	
H1	1.2440	0.7617	0.4861	0.074*	
C2	1.2999 (5)	0.6018 (2)	0.4538 (2)	0.0593 (6)	
H2	1.4410	0.6053	0.5076	0.071*	
C3	1.0170 (5)	0.50713 (19)	0.3160 (2)	0.0567 (6)	
H3	0.9552	0.4419	0.2700	0.068*	
C4	0.8953 (4)	0.60172 (17)	0.30288 (18)	0.0462 (5)	
C5	0.6677 (5)	0.59753 (18)	0.21628 (18)	0.0487 (5)	
C6	0.3816 (4)	0.72817 (18)	0.13538 (17)	0.0460 (5)	
C7	0.3225 (5)	0.83750 (18)	0.15148 (19)	0.0494 (5)	
C8	0.1218 (5)	0.8733 (2)	0.0871 (2)	0.0603 (6)	
H8	0.0841	0.9463	0.0990	0.072*	
C9	−0.0227 (5)	0.7994 (2)	0.0046 (2)	0.0630 (7)	
H9	−0.1591	0.8226	−0.0389	0.076*	
C10	0.0351 (5)	0.6921 (2)	−0.0129 (2)	0.0661 (7)	
H10	−0.0627	0.6430	−0.0686	0.079*	
C11	0.2372 (5)	0.6556 (2)	0.05113 (19)	0.0578 (6)	
H11	0.2759	0.5829	0.0378	0.069*	
C12	0.4185 (5)	1.01105 (19)	0.2637 (2)	0.0546 (6)	
C13	0.5372 (6)	1.1083 (2)	0.2146 (3)	0.0775 (8)	
H13	0.6570	1.1042	0.1620	0.093*	
C14	0.4776 (9)	1.2133 (3)	0.2440 (4)	0.1099 (15)	
H14	0.5563	1.2805	0.2105	0.132*	
C15	0.3048 (11)	1.2189 (4)	0.3215 (4)	0.130 (2)	
H15	0.2653	1.2897	0.3416	0.156*	
C16	0.1901 (10)	1.1207 (4)	0.3694 (4)	0.1308 (17)	
H16	0.0724	1.1248	0.4229	0.157*	
C17	0.2438 (7)	1.0151 (3)	0.3406 (3)	0.0896 (10)	
H17	0.1622	0.9478	0.3732	0.108*	
N1	0.9782 (4)	0.69785 (16)	0.36570 (16)	0.0559 (5)	
N2	1.2183 (4)	0.50575 (17)	0.39150 (18)	0.0621 (6)	
N3	0.5854 (4)	0.69918 (15)	0.20727 (15)	0.0500 (5)	
H3B	0.6696	0.7542	0.2519	0.060*	
O1	0.5723 (4)	0.50845 (13)	0.16236 (15)	0.0687 (5)	
O2	0.4781 (4)	0.90428 (14)	0.23700 (15)	0.0714 (6)	

### Atomic displacement parameters (Å^2^)

**Table:**

	*U*^11^	*U*^22^	*U*^33^	*U*^12^	*U*^13^	*U*^23^
C1	0.0680 (16)	0.0505 (14)	0.0679 (16)	0.0208 (12)	−0.0077 (14)	−0.0076 (12)
C2	0.0609 (15)	0.0574 (15)	0.0646 (16)	0.0240 (12)	0.0036 (13)	0.0053 (12)
C3	0.0668 (15)	0.0416 (12)	0.0660 (16)	0.0218 (11)	0.0043 (13)	−0.0005 (11)
C4	0.0525 (13)	0.0382 (11)	0.0518 (13)	0.0152 (9)	0.0123 (10)	0.0034 (9)
C5	0.0589 (14)	0.0368 (11)	0.0532 (13)	0.0147 (10)	0.0100 (11)	0.0004 (10)
C6	0.0503 (12)	0.0410 (11)	0.0474 (12)	0.0116 (9)	0.0024 (10)	0.0021 (9)
C7	0.0518 (13)	0.0415 (12)	0.0539 (13)	0.0097 (10)	−0.0021 (11)	−0.0021 (10)
C8	0.0623 (15)	0.0496 (13)	0.0693 (16)	0.0186 (12)	−0.0103 (13)	−0.0005 (12)
C9	0.0600 (15)	0.0663 (16)	0.0606 (16)	0.0147 (12)	−0.0130 (13)	0.0036 (13)
C10	0.0739 (17)	0.0612 (16)	0.0579 (15)	0.0090 (13)	−0.0118 (13)	−0.0076 (12)
C11	0.0726 (16)	0.0435 (12)	0.0562 (14)	0.0118 (11)	0.0003 (13)	−0.0039 (10)
C12	0.0604 (14)	0.0384 (11)	0.0650 (15)	0.0196 (10)	−0.0166 (12)	−0.0082 (10)
C13	0.0769 (19)	0.0607 (17)	0.089 (2)	0.0049 (14)	−0.0105 (16)	0.0104 (15)
C14	0.128 (3)	0.0379 (16)	0.143 (4)	0.0001 (18)	−0.072 (3)	0.0060 (19)
C15	0.176 (5)	0.072 (2)	0.146 (4)	0.079 (3)	−0.094 (4)	−0.056 (3)
C16	0.169 (4)	0.132 (4)	0.119 (3)	0.100 (3)	0.009 (3)	−0.029 (3)
C17	0.105 (2)	0.075 (2)	0.100 (2)	0.0375 (18)	0.029 (2)	0.0095 (17)
N1	0.0652 (13)	0.0419 (10)	0.0638 (12)	0.0212 (9)	−0.0005 (11)	−0.0027 (9)
N2	0.0707 (14)	0.0496 (11)	0.0725 (14)	0.0292 (10)	0.0020 (12)	0.0035 (10)
N3	0.0595 (12)	0.0378 (10)	0.0541 (11)	0.0168 (8)	−0.0027 (9)	−0.0043 (8)
O1	0.0871 (13)	0.0430 (9)	0.0761 (12)	0.0212 (9)	−0.0087 (10)	−0.0118 (8)
O2	0.0782 (12)	0.0495 (10)	0.0877 (13)	0.0331 (9)	−0.0328 (10)	−0.0241 (9)

### Geometric parameters (Å, º)

**Table:**

C1—N1	1.328 (3)	C9—C10	1.371 (3)
C1—C2	1.378 (3)	C9—H9	0.9300
C1—H1	0.9300	C10—C11	1.387 (3)
C2—N2	1.327 (3)	C10—H10	0.9300
C2—H2	0.9300	C11—H11	0.9300
C3—N2	1.329 (3)	C12—C17	1.353 (4)
C3—C4	1.385 (3)	C12—C13	1.357 (4)
C3—H3	0.9300	C12—O2	1.391 (3)
C4—N1	1.333 (3)	C13—C14	1.379 (5)
C4—C5	1.501 (3)	C13—H13	0.9300
C5—O1	1.221 (3)	C14—C15	1.355 (6)
C5—N3	1.349 (3)	C14—H14	0.9300
C6—C11	1.390 (3)	C15—C16	1.352 (7)
C6—C7	1.393 (3)	C15—H15	0.9300
C6—N3	1.407 (3)	C16—C17	1.371 (5)
C7—C8	1.380 (3)	C16—H16	0.9300
C7—O2	1.390 (3)	C17—H17	0.9300
C8—C9	1.382 (3)	N3—H3B	0.8600
C8—H8	0.9300		
			
N1—C1—C2	122.8 (2)	C11—C10—H10	119.5
N1—C1—H1	118.6	C10—C11—C6	119.7 (2)
C2—C1—H1	118.6	C10—C11—H11	120.2
N2—C2—C1	121.5 (2)	C6—C11—H11	120.2
N2—C2—H2	119.2	C17—C12—C13	121.5 (3)
C1—C2—H2	119.2	C17—C12—O2	118.3 (2)
N2—C3—C4	122.7 (2)	C13—C12—O2	120.3 (3)
N2—C3—H3	118.6	C12—C13—C14	119.0 (3)
C4—C3—H3	118.6	C12—C13—H13	120.5
N1—C4—C3	121.0 (2)	C14—C13—H13	120.5
N1—C4—C5	118.89 (18)	C15—C14—C13	120.2 (4)
C3—C4—C5	120.1 (2)	C15—C14—H14	119.9
O1—C5—N3	125.8 (2)	C13—C14—H14	119.9
O1—C5—C4	121.02 (19)	C16—C15—C14	119.5 (3)
N3—C5—C4	113.19 (18)	C16—C15—H15	120.2
C11—C6—C7	118.6 (2)	C14—C15—H15	120.2
C11—C6—N3	124.6 (2)	C15—C16—C17	121.3 (4)
C7—C6—N3	116.74 (19)	C15—C16—H16	119.3
C8—C7—O2	124.1 (2)	C17—C16—H16	119.3
C8—C7—C6	121.3 (2)	C12—C17—C16	118.4 (4)
O2—C7—C6	114.61 (19)	C12—C17—H17	120.8
C7—C8—C9	119.4 (2)	C16—C17—H17	120.8
C7—C8—H8	120.3	C1—N1—C4	116.03 (19)
C9—C8—H8	120.3	C2—N2—C3	115.9 (2)
C10—C9—C8	120.0 (2)	C5—N3—C6	129.29 (19)
C10—C9—H9	120.0	C5—N3—H3B	115.4
C8—C9—H9	120.0	C6—N3—H3B	115.4
C9—C10—C11	121.0 (2)	C7—O2—C12	118.20 (17)
C9—C10—H10	119.5		
			
N1—C1—C2—N2	0.0 (4)	C13—C14—C15—C16	−0.3 (6)
N2—C3—C4—N1	0.8 (4)	C14—C15—C16—C17	−0.4 (6)
N2—C3—C4—C5	−179.7 (2)	C13—C12—C17—C16	−0.7 (5)
N1—C4—C5—O1	−174.6 (2)	O2—C12—C17—C16	178.2 (3)
C3—C4—C5—O1	6.0 (3)	C15—C16—C17—C12	0.9 (6)
N1—C4—C5—N3	4.8 (3)	C2—C1—N1—C4	0.1 (4)
C3—C4—C5—N3	−174.7 (2)	C3—C4—N1—C1	−0.4 (3)
C11—C6—C7—C8	1.2 (4)	C5—C4—N1—C1	−179.9 (2)
N3—C6—C7—C8	−178.5 (2)	C1—C2—N2—C3	0.4 (4)
C11—C6—C7—O2	−179.6 (2)	C4—C3—N2—C2	−0.8 (4)
N3—C6—C7—O2	0.7 (3)	O1—C5—N3—C6	−1.3 (4)
O2—C7—C8—C9	−179.4 (2)	C4—C5—N3—C6	179.3 (2)
C6—C7—C8—C9	−0.3 (4)	C11—C6—N3—C5	−5.7 (4)
C7—C8—C9—C10	−0.4 (4)	C7—C6—N3—C5	174.0 (2)
C8—C9—C10—C11	0.1 (4)	C8—C7—O2—C12	3.9 (4)
C9—C10—C11—C6	0.8 (4)	C6—C7—O2—C12	−175.3 (2)
O2—C12—C13—C14	−179.0 (2)	C17—C12—O2—C7	91.4 (3)
C12—C13—C14—C15	0.6 (5)	C13—C12—O2—C7	−89.7 (3)

### Hydrogen-bond geometry (Å, º)

**Table:**

*D*—H···*A*	*D*—H	H···*A*	*D*···*A*	*D*—H···*A*
C2—H2···N2^i^	0.93	2.62	3.439 (3)	147

Symmetry code: (i) −*x*+3, −*y*+1, −*z*+1.
